# Spontaneous humeral torsion deformity correction after displaced supracondylar fractures in children

**DOI:** 10.1186/s12891-021-04909-y

**Published:** 2021-12-06

**Authors:** Anna K. Hell, Claudia Gadomski, Lena Braunschweig

**Affiliations:** Pediatric Orthopaedics; Department of Trauma, Orthopaedic and Plastic Surgery, University Medical Center Goettingen, 37075 Goettingen, Germany

**Keywords:** Supracondylar humeral fractures, Torsion, Cubitus varus, Humeral torsion, Rotation

## Abstract

**Background:**

After displaced supracondylar humerus fractures (SCHF) in children, residual deformities are common with cubitus varus (CV) being the clinically most visible. Distal fragment malrotation may lead to instability, fragment tilt and subsequent CV. Detection and assessment of malrotation is difficult and the fate of post-traumatic humeral torsion deformity is unknown. The aim of this study was to evaluate the incidence of humeral torsion differences in children with surgically treated SCHF and to observe spontaneous changes over time.

**Methods:**

A cohort of 27 children with displaced and surgically treated SCHF were followed prospectively from the diagnosis until twelve months after trauma. Clinical, photographic, sonographic and radiological data were obtained regularly. Differences in shoulder and elbow motion, elbow axis, sonographic humeral torsion measurement and radiological evaluation focusing on rotational spur were administered.

**Results:**

Six weeks after trauma, 67% of SCHF children had a sonographically detected humeral torsion difference of > 5° (average 14.0 ± 7.6°). Of those, 44% showed a rotational spur, slight valgus or varus on radiographs. During follow-up, an average decrease of the difference from 14° (six weeks) to 7.8° (four months) to 6.5° (six months) and to 4.9° (twelve months) was observed. The most significant correction of posttraumatic humeral torsion occurred in children < 5 years and with internal malrotation > 20°.

**Conclusion:**

After displaced and surgically treated SCHF, most children had humeral torsion differences of both arms. This difference decreased within one year after trauma due to changes on the healthy side or correction in younger children with severe deformity.

**Level of Evidence/Clinical relevance:**

Therapeutic Level IV

## Background

Supracondylar humeral fractures (SCHF) are the most common pediatric elbow fractures [1]. The majority of these fractures are extension-type ones as the result of falling on to the outstretched hand with the elbow extended. Diagnosis is based on clinical evaluation and conventional radiography. Several classifications [2–6] focus on fracture stability versus instability and bone contact versus displacement. Unstable and/or displaced fractures are usually treated by reduction and internal fixation. Currently, the first choice of treatment is closed reduction and percutaneous crossed pin fixation, a surgical method with reliable biomechanical testing and low loss of reduction [7–10]. Complications associated with supracondylar humeral fractures are not uncommon and include neurovascular lesions, reduced range of motion, compartment syndrome and cubitus varus (CV) deformity [11, 12].

In the majority of cases, CV is a combination of varus, hyperextension and internal rotation [13]. Distal fragment malrotation may lead to instability, fragment tilt and subsequent varus of the elbow joint. CV not only causes poorly tolerated cosmetic deformity of the elbow but might also increase the risk of lateral condyle fractures, internal rotational malalignment, pain, malfunction of the elbow and other secondary fractures [14, 15].

There has been no simple clinical method of measuring humeral torsion deformity. Shoulder function in children is too variable and dependent on muscle tone to allow reliable conclusions concerning humeral torsion. The desire for a measuring device is old [16] and various measurement methods for determining the torsion angle of the humerus can be found in the literature: anatomical, radiographic, with computed tomography (CT) or magnetic resonance imaging (MRI) and ultrasound measurements. All these measurement methods and guidelines for determining humeral torsion are highly variable and lead to differences in the torsional angle values for adults reported in the literature [17–21]. However, the correlation between most of these methods was highly significant with a correlation coefficient between 0.79 and 0.83 [17, 19, 22].

As described by Krahl in 1947, humeral torsion is age dependent similar to femoral anteversion [20]. The torsion of the humerus develops reversely in utero than in children and adolescents. Changes are most likely caused by the age-dependent position of the scapula, the shape of the thorax and muscle forces [20, 23]. In his cadaver study, Krahl reported a cessation of torsion at times of proximal epiphyseal closure, which suggest growth-dependent changes [20].

The aim of this prospective cohort study was to evaluate the incidence of humeral torsion differences (e.g. rotation deformity) in children with displaced and surgically treated SCHF as well as to observe spontaneous changes over time using ultrasound.

## Material and methods

After ethics committee approval, children with displaced SCHF and surgical treatment, which were primarily seen and treated at a University Pediatric Trauma Centre, were prospectively recruited and families were informed about the purpose of the study, which was to perform rotational sonographic measurements in addition to standardized follow-up appointments. All children received closed fracture reduction and crossed K-wire fixation.

**Fig. 1:**
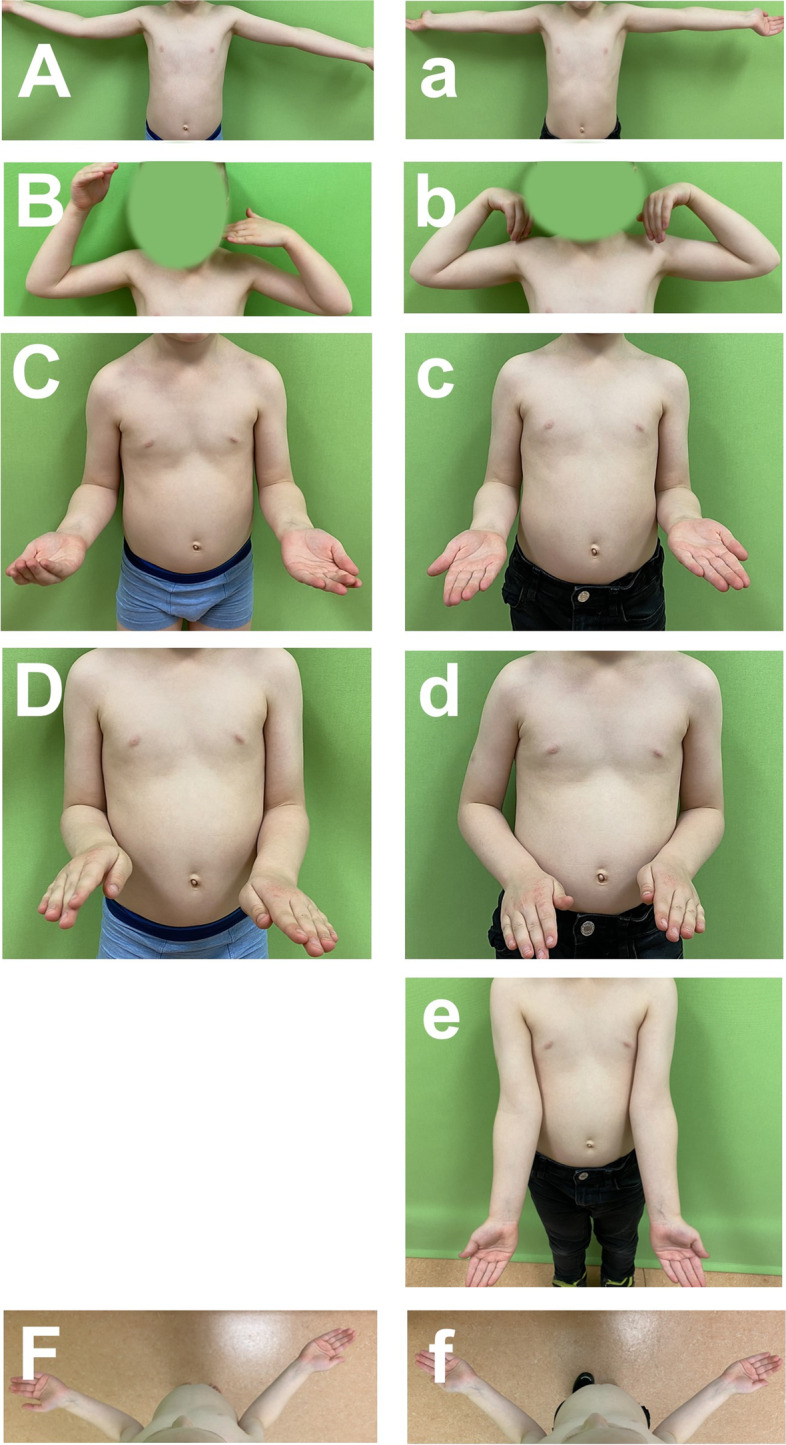
Photographic documentation of the elbow status at six weeks (A-F) and 12 months follow-up (a-f): extension (A,a), flexion (B,b), supination (C,c), pronation (D,d), elbow axis (e) and external shoulder rotation (F,f). Six weeks after trauma, elbow axis was not determined because of an extension deficit

A cohort of 27 children was followed from the time of injury until twelve months after the accident. Clinical, photographic (Fig. 1) and sonographic data were documented four times: six weeks, four months, six months and twelve months after the accident. Radiographs of the elbow in two planes were performed at the initial presentation at the day of injury, after surgery, three weeks after surgery before removal of metal and at the one-year follow-up. Radiographic evaluation consisted of the anterior humeral line (AHL; Roger’s line) [24, 25] on lateral radiographs as well as valgus and varus evaluation three weeks after surgery. Rotational deformity was defined if a rotational spur was visible ventral of the AHL on lateral radiographs or if an axis misalignment on anterior posterior (a.p.) views was present. At radiological one-year follow-up, remodeling was evaluated.

At each follow-up, Flynn criteria were administered [26]. Patients and parents were asked about pain, functional disability or cosmetic impairment. The range of motion of the upper extremity for the shoulder and elbow joint was determined and documented for both arms using the neutral zero method. The elbow arm axis was only graded if full elbow extension was restored. Range of motion was compared between the injured and non-injured arm and differences were calculated. To minimize measurement errors, only clinical differences ≥5° were graded as deviations. Range of motion was photo-documented (Fig. 1).

**Fig. 2:**
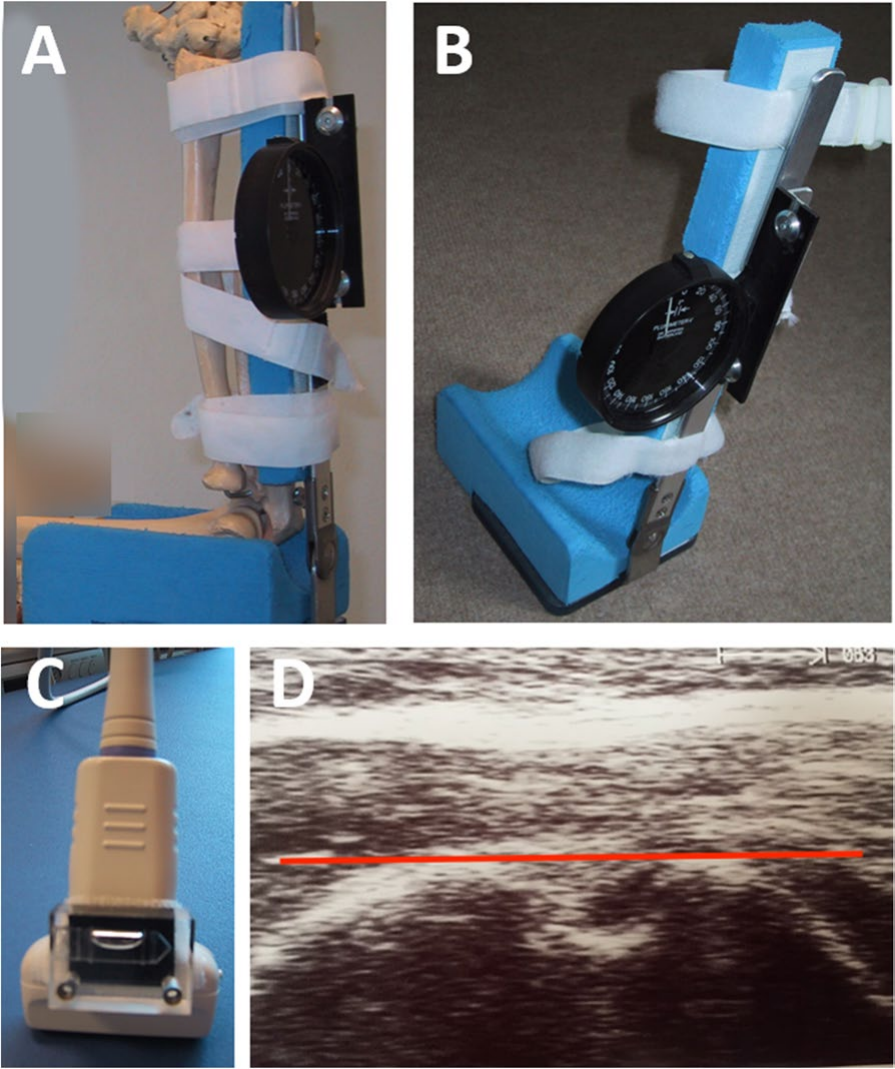
90° elbow flexion and positioning of the arm in the measuring device (A,B) The model of a skeleton is used to illustrate the positioning of the bones of the forearm. A spirit level was installed on the linear ultrasound probe (C). Sonographic picture of the sulcus bicipitis in a horizontal alignment (D). Humeral torsion value was read on the angular degree measuring device (A,B)

Sonographic evaluations were done using an Aloka® sonography device (SSD-1700 DynaView II; Aloka Co., Ltd. Europe Office, 2143 MZ Hoofddrop, The Netherlands) and a 7.5 MHz linear transducer. The elbow of the examined arm was flexed in a 90° bending with the patient lying supine and fixated in a positioning device (Fig. 2a,b) which blocked movement of the elbow joint [27]. On the linear ultrasound probe a spirit level was installed (Fig. 2c) and the ultrasound probe was used to locate the intertubercular sulcus on the proximal humeral head (Fig. 2d). For this purpose, the ultrasound head was placed perpendicular to the longitudinal axis of the humerus. By rotating the arm, the major and minor tubercles were displayed so that they were aligned horizontally in the ultrasound display on the screen with a balanced spirit level. They formed a 90 ° angle with a vertical line through the lowest point of the intertubercular sulcus (Fig. 2d). The humeral torsion was determined by the value of the angular degree measuring device, which was mounted on the bearing shell.

All measurements were done by two independent investigators to enable evaluation of inter-observer errors. Prior to this investigation, this sonographic method was validated by sonographic torsion measurements of 122 extremities carried out by two independent investigators at different times [25] with a possible measurement error of 3.6° (mean + standard deviation; reliability 0.984 left and 0.986 right arm, Cronbach alpha).

The data were analyzed statistically with Student’s t-test or repeated measures ANOVA with Bonferroni correction using GraphPad Prism version 4.0 (San Diego, CA, USA). Statistical significance was defined with the *p*-value at 0.05 or lower (*p* < 0.05).

## Results

A complete data set of clinical, sonographic and radiographic evaluations of 27 children (16 males, 11 females) with a displaced supracondylar humeral fracture was evaluated. The mean age at trauma was 6.8 years (SD = 2.6). The main causes for the fracture were unobserved falls, followed by falls during sporting activities. Mainly the right side was affected (*n* = 18). Almost all children (*n* = 26) were surgically treated on the day of the accident, whereas one patient with secondary displacement was treated on the sixth day after trauma.

**Fig. 3:**
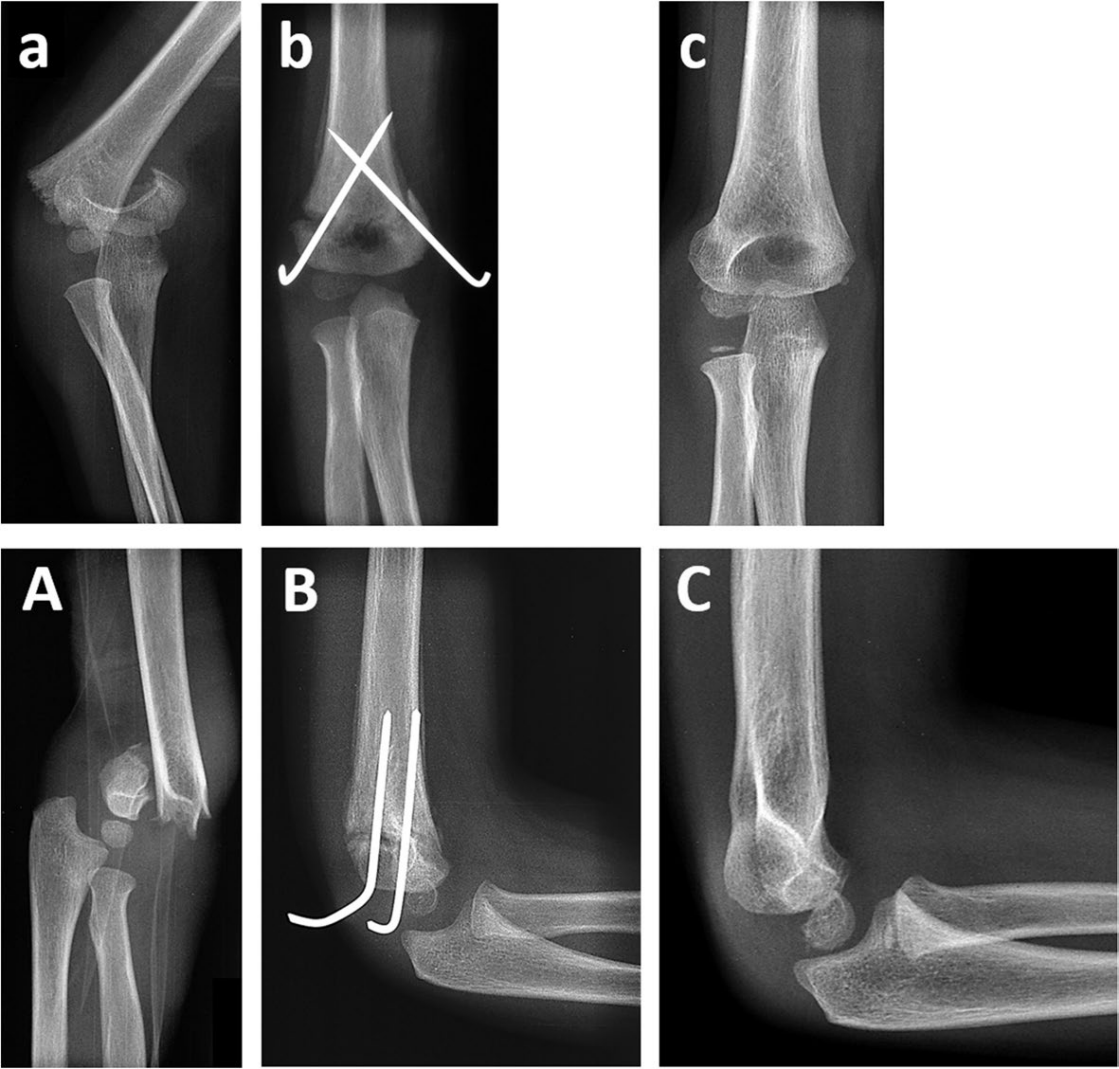
A.p. (a) and lateral (A) elbow radiographs of a displaced SCHF in a five-year old boy, which was treated surgically by closed reduction and crossed K-wire fixation. Consolidation radiographs after three weeks (B,b) showed a small remaining varus deformity on the a.p. view (b) and no rotational deformity laterally (B). At one year follow-up complete remodeling has occurred except for an indentation of the lateral distal humerus contour (C,c)

All fractures were treated by closed reduction and percutaneous crossed K-wire fixation (Fig. 3), followed by a plaster immobilization for an average of 25.5 days (range 21 to 37 days). After plaster removal, a consolidation X-ray of the elbow in two planes was taken and the crossed K-wires were removed in an outpatient setting without anesthesia. All children spontaneously moved the injured limb. None of the patients received physiotherapy. Four complications occurred. Three patients had pin infections, which were treated with antibiotics. One patient had loosened wires which required surgical revision to ensure the original positioning.

At all follow-up examinations, patients denied pain or cosmetic impairment. However, functional limitations were observed by all patients at the six-week follow-up. After four months, only five children still experienced functional limitations which again was reduced to only one patient after six months after the accident. At the last follow-up after one year, none of the patients observed limitations in the function of the arm. No physiotherapy was administered.

On clinical examination, internal and external shoulder rotation significantly (*p* < 0.01) differed to the unaffected side with more internal and less external rotation (Table 1). These findings probably reflect more internal rotational deformity than external humeral torsion.

**Table 1:** Shoulder and elbow function, elbow axis and sonographically determined humeral torsion after displaced and surgically treated SCHF in children

		6 weeks	4 months	6 months	12 months
internal rotation shoulder	healthy fractured	76.1 ± 10.6 ° 79.3 ± 11.4 ° (*p* > 0.05)	76.7 ± 10.4 ° 78.2 ± 11.1 ° (*p* > 0.05)	77.1 ± 9.4 ° 79.6 ± 10.4 ° (*p* > 0.05)	76.3 ± 10.1 ° 80.9 ± 8.8 ° **(*p*** **< 0.05)**
external rotation shoulder	healthy fractured	105.0 ± 12.0 ° 98.5 ± 11.6 ° **(***p* **< 0.05)**	104.4 ± 11.2 ° 100.4 ± 12.2 ° **(*p*** **< 0.05)**	105.6 ± 12.2 ° 102.9 ± 11.3 ° (*p* > 0.05)	105.7 ± 11.3 ° 101.3 ± 11.7 ° **(*p*** **< 0.05)**
elbow extension	healthy fractured	- 11.9 ± 4.2 ° 28.3 ± 20.9 ° **(*p*** **< 0.05)**	- 12.6 ± 4.0 ° 1.5 ± 12.2 ° **(*p*** **< 0.05)**	- 11.9 ± 3.8 ° − 5.8 ± 9.3 ° **(*p*** **< 0.05)**	- 12.4 ± 3.2 ° − 10.6 ± 4.7 ° **(*p*** **< 0.05)**
elbow flexion	healthy fractured	140,9 ± 4,6 ° 126.9 ± 10.2 ° **(*p*** **< 0.05)**	140.7 ± 3.6 ° 133.3 ± 9.2 ° **(*p*** **< 0.05)**	140.8 ± 3.7 ° 137.1 ± 7.4 ° **(*p*** **< 0.05)**	141.7 ± 3.9 ° 140.9 ± 4.4 ° (*p* > 0.05)
elbow axis	healthy fractured	- -	−8.5 ± 2.9 ° − 8.5 ± 3.3 ° (*p* > 0.05)	−8.5 ± 3.3 ° − 6.5 ± 4.6 ° **(*p*** **< 0.05)**	−8.5 ± 3.7 ° − 6.5 ± 5.6 ° (*p* > 0.05)
humerus torsion	healthy fractured	20.9 ± 14.8 ° 28.4 ± 11.0 ° **(*p*** **< 0.05)**	22.3 ± 13.7 ° 27.4 ± 10.0 ° **(*p*** **< 0.05)**	23.7 ± 12.7 ° 27.7 ± 9.2 ° **(*p*** **< 0.05)**	24.9 ± 12.2 ° 28.1 ± 9.5 ° **(*p*** **< 0.05)**

Elbow extension and flexion deficits improved significantly (*p* < 0.05) during follow-up and showed only a marginal difference to the healthy arm after twelve months. The elbow axis was determined if full extension had been reached. Six weeks after trauma, free extension had not yet been restored in 21 cases. Therefore, data are only shown for the other three time points with no significant changes except for a minor difference after six months.

The sonographic examination of the humeral torsion showed a significant difference in all follow-up examinations between the fractured and the healthy arm. This difference was greatest six weeks after trauma and was reduced during follow-up and individual growth. The absolute values of the fractured arm did not change significantly during the 12 months follow-up.

Even though the analysis of the inter-observer error of sonographic measurements of humeral torsion showed a very high correlation (0.97) between both investigators, there was an average measurement difference of 3.6° using the described ultrasound method. Therefore, a sonographically detected difference in humeral torsion ≤5° was assessed as no difference to the healthy side. Using these criteria, 33% (*n* = 9) children had no humeral torsion difference and this finding remained stable during follow-up (Table 2). Six weeks after the accident, 67% (*n* = 18) of patients had a difference (average 14.0 ± 7.6 °) in sonographically measured humeral torsion, which was significantly (*p* < 0.05) reduced between 6 weeks and 4 months but remained identical thereafter (Table 2).

**Table 2:** Sonographically measured humeral torsion differences in comparison to the healthy arm in children with displaced and surgically treated SCHF [* *p* < 0.05]

Humeral torsion difference	6 weeks	4 months	6 months	12 months
None (difference ≤ 5°) (*n* = 9; 33%)	1.4 ± 1.9 °	1.1 ± 1.7 °	2.4 ± 2.9 °	0.8 ± 1.4 °
Present (difference > 5°) (*n* = 18; 67%)	14.0 ± 7.6 ° *	7.8 ± 8.4 ° *	6.5 ± 7.6 °	4.9 ± 6.6 °

To analyze the effect of the patients’ age on humeral torsion difference, three age groups were formed: < 5 years, 5–10 years and > 10 years. These age groups were analyzed depending on the difference in humeral torsion and direction (e.g. internal or external humeral torsion). Even though this division in subgroups resulted in small individual numbers, correction potential was highest in children < 5 years with severe internal rotational deformity > 20° (*n* = 2; changes in humeral torsion difference 15°). Humeral torsion changes did not correlate to the probably more used dominant arm.

To correlate sonographically determined humeral torsion and radiographic findings, consolidation radiographs taken on an average 25.5 days (range 21 to 37 days) after surgery before removal of K-wires were analyzed. A rotational spur or axis malalignment were found in two children (22%) of the group without sonographically detected humeral torsion (*n* = 9), while 44% (*n* = 8) of cases in the pathological humeral torsion group (*n* = 18) had radiological abnormalities. These consisted of five rotation spurs, one cubitus varus, one cubitus valgus and one dorsal tip. In this group, the average torsional difference was 14°. Radiologically, complete bone remodeling was present one year after trauma.

## Discussion

Displaced supracondylar fractures of the humerus (SCHF) in children are often accompanied by a number of complications. Elbow function is usually restored within twelve months after the accident [12, 28], which is consistent with findings in this study. Contrary to this, residual cubitus varus (CV) is a persistent problem. In the majority of cases, CV usually is a combination of varus, hyperextension and internal rotation [13]. While Hindman et al. described an association with CV and a torsion error over 10° [29], other authors [30–33] were able to demonstrate CV misalignments without torsion differences. Analyzing 3D bone models created from computed tomography data, Takeyasu et al. found bony deformity in addition to CV in 80% of cases and isolated CV in 20% [13]. While these data clearly show that CV is often associated with humeral torsion deformity probably reflecting fracture instability due to reduced bone contact at the level of fracture, the remodeling potential of posttraumatic humeral torsion deformity after SCHF in children remains unclear.

Various methods of detecting humeral torsion have been described in the past. The clinical measurement of the shoulder function [30, 34, 35] as an indirect indication of humeral torsion has proven to be rather inaccurate, which was confirmed in the present study. However, clinical measurements will point in the right direction in cases with severe pathology. Radiographic diagnosis as a method to detect humeral torsion over time is difficult. In the early course after the accident, a rotational ventral spur can be seen on lateral radiographs as a clear sign of rotational deformity. In our collective, a total of 37% of the children showed a rotation spur on lateral radiographs and/or malalignement at the time of fracture consolidation three weeks after the accident. In the literature, up to 47% of cases showed rotation spurs when evaluating radiological images [5]. However, depending on the beam path in the lateral radiographic image, torsional errors of up to 20° remain undetected [36]. There are some studies on estimation of rotational deformity by quantification of the ventral spur, but overall estimation remains difficult [36–39]. Over time, a ventral rotational spur usually remodels and is therefore no longer detectable on radiographs. Changes in humeral torsion during growth cannot be evaluated using plain radiography. Using repetitive CT examinations for assessment of humeral torsion in children is difficult because of excessive radiation and repetitive MRI investigations are time consuming and may require sedation in young children. Common to all methods is that standardization is difficult due to the lack of concise orientation points of the humerus and dynamic development of humeral torsion during growth [20]. Sonographic examinations are quickly available, non-invasive, inexpensive and easily performed. This method seems to be ideal to record humeral torsion and has been used for this purpose in the past [19, 27, 40]. However, a major problem presents the lack of distinct measurement points of the proximal humerus. In this paper, the applied method overcomes this problem by using a standardized positioning device with 90° elbow flexion and proximal humerus orientation with a spirit level installed on the linear ultrasound probe. Administering this method, Katthagen et al. were able to evaluate humeral torsion in hemiplegic children [27]. Also, the measurement error of this sonographic method was 3.6° and therefore lower than in previous studies [40].

Using this sonographic method, rotational deformity after displaced and surgically treated SCHF in children was found in 67% with an average of 14° difference six weeks after the accident. A significant change of humeral torsion difference could be detected between the six week and four months follow-up. Despite small numbers, changes were most profound in younger children and with severe internal humeral torsion deformity.

As described by Krahl in 1947, there is a physiological change in humeral torsion during growth [20], which was confirmed by the sonographic measurements in our study. A physiological increase towards more internal rotation in humeral torsion was found on the healthy arm with advancing age. Most post-traumatic rotational deformities of the humerus resulted in an increased internal rotation, which has been described in the literature before [13]. The collected data in this paper suggest that both values approximated again in the course of time, i.e. there was a significant reduction in the difference. Such phenomena have been reported after post-traumatic rotational deformity of the femur in children [41].

Despite these interesting results, there are limitations to this study. A main limitation is that the number of patients is low and results might be underpowered. Further evaluation in a larger population might be interesting. The values of humeral torsion both of the healthy and fractured side showed a wide range indicating large individual variance. Larger studies for age-dependent normal values are necessary.

Whether athletic activity or muscle training can influence development of torsion after SCFH in children was not considered in this study. Such mechanism was described by Pieper for handball players [22]. Even though sonographic measurement of humeral torsion in children seems to be reliable using the described method, sonographic results do not correlate to clinical and radiological findings in some cases.

## Conclusions

To our knowledge for the first time, this paper presents data on spontaneous rotational deformity correction in children with surgically treated displaced SCHF, which mainly occurred within the first months after trauma. The time period of deformity correction and higher correction potential at a younger age suggest a mixture of a functional-mechanical correction mechanism, as is also known, for example, for the spontaneous correction of the displaced proximal radius fractures in children [42], in combination with a correction due to growth. An analysis of post-traumatic humeral torsion differences in adults and their development over time would be interesting to differentiate between these mechanisms.

## Abbreviations

AHL: anterior humeral line
ANOVA: analysis of variance
a.p.: anterior posterior
CT: computed tomography
CV: cubitus varus
SCHF: supracondylar humerus fractures

## Declarations

All methods were performed in accordance with the relevant guidelines and regulations.
